# Precise irrigation water and nitrogen management improve water and nitrogen use efficiencies under conservation agriculture in the maize-wheat systems

**DOI:** 10.1038/s41598-023-38953-6

**Published:** 2023-07-26

**Authors:** Naveen Gupta, Yadvinder Singh, Hanuman S. Jat, Love K. Singh, Kajod M. Choudhary, Harminder S. Sidhu, Mahesh K. Gathala, Mangi L. Jat

**Affiliations:** 1 Borlaug Institute for South Asia (BISA), CIMMYT, Ladhowal, Punjab 141004 India; 2 ICAR-Central Soil Salinity Research Institute (CSSRI), Karnal, 132001 India; 3 International Maize and Wheat Improvement Centre (CIMMYT), Dhaka, Bangladesh; 4 International Maize and Wheat Improvement Centre (CIMMYT), NASC Complex, Pusa, New Delhi, 110012 India; 5 Present Address: Punjab Agricultural University, Ludhiana, Punjab 141004 India; 6 Present Address: International Crops Research Institute for the Semi-Arid Tropics (ICRISAT), Hyderabad, India

**Keywords:** Plant sciences, Climate sciences, Environmental sciences

## Abstract

A 3-year field experiment was setup to address the threat of underground water depletion and sustainability of agrifood systems. Subsurface drip irrigation (SDI) system combined with nitrogen management under conservation agriculture-based (CA) maize-wheat system (MWS) effects on crop yields, irrigation water productivity (WP_i_), nitrogen use efficiency (NUE) and profitability. Grain yields of maize, wheat, and MWS in the SDI with 100% recommended N were significantly higher by 15.8%, 5.2% and 11.2%, respectively, than conventional furrow/flood irrigation (CT-FI) system. System irrigation water savings (~ 55%) and the mean WP_i_ were higher in maize, wheat, and MWS under the SDI than CT-FI system. There was saving of 25% of fertilizer N in maize and MWS whereas no saving of N was observed in wheat. Net returns from MWS were significantly higher (USD 265) under SDI with 100% N (with no subsidy) than CT-FI system despite with higher cost of production. The net returns were increased by 47% when considering a subsidy of 80% on laying SDI system. Our results showed a great potential of complementing CA with SDI and N management to maximize productivity, NUE, and WP_i_, which may be economically beneficial and environmentally sound in MWS in Trans-IGP of South Asia.

## Introduction

The maize-wheat system is the third most important cropping system (~ 2.90 M ha) after the rice–wheat and cotton-wheat systems^1^ that has potential in view of an emerging water crisis in the Indo-Gangetic plains of South Asia. Under conventional flood irrigation in rice, a major amount of irrigation water is lost through evaporation and soil percolation^2,3^. In recent years, the area under maize cultivation has increased in north-west (NW) India largely due to favorable government policies for promoting its cultivation to save precious irrigation water and electricity costs^4^. Growing conventional maize and wheat on a flat field requires 6–7 tillage operations along with flood irrigation, which involves high energy input and inefficient use of irrigation water and fertilizer nitrogen (N), and in less economic profits^5^. An alternate technique to save water and increase irrigation water productivity (WP_i_) and fertilizer nitrogen use efficiency (NUE) comprises a shift to permanent raised beds based conservation agriculture (CA) practices (no-tillage and straw mulching), irrigation scheduling and fertigation^1,6^.

There is a great challenge to enhance productivity and decrease cultivation costs in the maize-wheat system. The adoption of furrow-irrigated permanent raised beds increased yields, NUE, addressed labor and water issues and saved the environment^7–10^. In a permanent raised bed planting system, both maize and wheat are planted on the top of 37.5 cm wide beds with 30 cm wide furrows and retaining previous crop residues on the soil surface^11^. Previous studies showed that maize planted on permanent raised beds with retention of residues resulted in 11% savings in irrigation water and 16% higher WP_i_ compared to conventional planting^8,12^.

Conservation agriculture coupled with precise water and nutrient application shall provide opportunities for an intensification of cereal systems for a much needed change towards transforming agrifood systems in South Asia. The layering of drip irrigation combined with N fertigation in the CA system could be an economically feasible choice for field row crops, such as maize^13–15^ and wheat^15–18^ to increase WP_i_ and NUE. Surface drip irrigation has a serious limitation due to the complex process of anchoring laterals at the start and removing them after the harvest of each crop because they interfere with field operations during the year. Unlike surface drip irrigation, the subsurface drip irrigation (SDI) system reduces soil evaporation, permits better delivery of water and fertilizers directly to the plant root zone to meet and synchronize the plant demand which results in higher WP_i_ and NUE, and it also saves labor cost and allows normal tillage practices^13,18,19^. Conventional surface application of N as urea encourages volatilization losses of N particularly when crop residues are retained as mulch^20,21^. Precise N fertigation in several splits through SDI might reduce N losses via leaching and volatilization, thereby improving NUE in a CA-based permanent raised bed maize-wheat system^19,22,23^. Thus precise N placement along with irrigation water in the active crop root zone via SDI will enhance NUE and WP_i_ in a CA-based permanent raised bed maize-wheat system.

Limited information is available on the effect of SDI systems on yield, water productivity and NUE on the CA-based annual maize-wheat system. Pablo et al.^24^ and Douh and Boujelben^25^ reported higher WP_i_ under SDI compared to flood irrigation in maize. Jat et al.^19^ evaluated the potential of SDI under a CA-based maize-wheat-mung bean (*Vigna radiata*) system using flat-bed planting on partially reclaimed soil and reported significant increases in WP_i_ and NUE under SDI compared to conventional flood irrigated systems. However, their study lacks information on the crop responses of maize and wheat to varying N rates applied through SDI under a permanent raised bed system. Therefore, detailed scientific information is urgently needed on the effect of the SDI method on N response, WP_i,_ and NUE in the regions of severe water scarcity and injudicious use of N fertilizer in CA-based permanent raised bed maize-wheat system in NW India. We hypothesized that CA-based practices (permanently raised bed planting and straw mulching) layered with SDI system and N fertigation at different levels will increase crop yields, WP_i,_ and NUE in the maize-wheat system. The objective of our study was to evaluate the effects of N levels on crop yields, irrigation water savings, WP_i_, NUE, and economics under SDI in a CA-based irrigated maize-wheat system in NW India.

## Results

### Weather

#### Maize season

**Figure 1:**
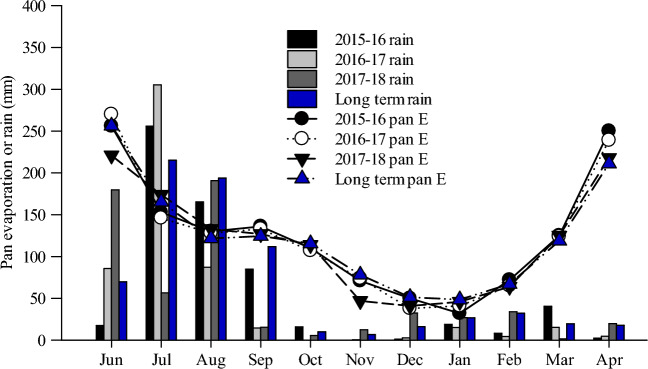
Monthly mean total pan evaporation and rainfall during 2015–16, 2016–17, 2017–18 and the long-term averages (1970–2014).

**Figure 2:**
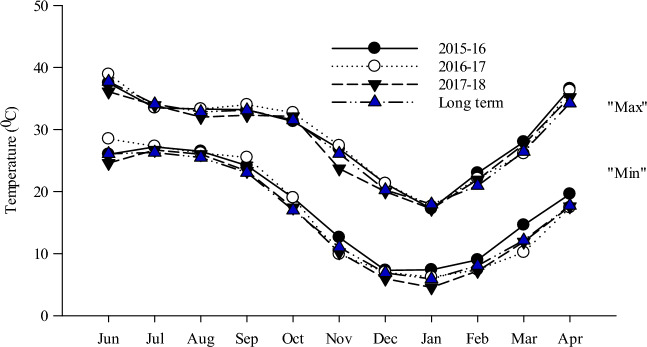
Monthly mean maximum and minimum temperatures during 2015–16, 2016–17, 2017–18 and the long-term averages (1970–2014).

The precipitation for the years 2015 (541 mm), 2016 (494 mm), and 2017 (444 mm) in maize seasons were lesser than the long-term average (LTA) (June-October) of 603 mm (Fig. 1). Its distribution of pattern was also quite dissimilar during the three seasons, with low rains in June 2015, July 2017, August 2016 and 2017, and September 2017, whereas quite high rains were recorded during July 2017. Monthly pan evaporation (E) during three maize seasons was generally comparable to the LTA (Fig. 1). Total pan E in June-October 2015 (785 mm), 2016 (784 mm), and 2017 (769 mm) was comparable to that of LTA (784 mm). The minimum monthly temperature was 1–2 °C higher than the LTA throughout the growing season in 2015 and 2016 (Fig. 2). The maximum monthly temperature was normally the same as the LTA, except for a higher value in July 2016.

#### Wheat season

Total rainfall during the 2015–16 and 2016–17 wheat seasons (November–April) was 74 and 46 mm, respectively, (Fig. 1), which was lower than the LTA of 122 mm. However, in 2017–18 total rainfall (130 mm) was matching to the LTA. Total pan E during the wheat season was 600, 581 and 541 mm in 2015–16, 2016–17, and 2017–18, respectively, compared to the LTA (575 mm) (Fig. 1). Mean monthly Max and Min T was higher than the LTA during the 3 years of study, apart from higher Min T was recorded in March–April 2016, and lower Max T in March 2017 (Fig. 2).

### Grain yield and yield attributes

Significant interaction effects of year × treatments were observed for grain yields of maize and wheat crops, therefore year-wise yield data are discussed in the following section.

### Maize

Maize grain yield declined over the years irrespective of the treatments. It was significantly higher in 2015 (7.73 t ha^–1^) compared to 2016 (6.95 t ha^–1^) and 2017 (6.53 t ha^–1^). Maize yield increased significantly with increasing N rate up to 100% of recommended N rate (T4) in the SDI system in all three years of the study. Mean grain yield (averaged 3-years) with T4 was higher by 132% compared to no-N control (T1) (Table 1). At the recommended N rate (100% N), the 3-yr mean data (average over 3-yr) indicated that maize yield was significantly higher (16.0%) in SDI (T4) compared to conventional FI system (T5) (Table 1). The grain yield of maize (3-yr mean) with 75% of recommended N dose in the SDI (T3) system was similar to that with 100% N in the conventional FI system, thereby helping in saving 25% of N fertilizer. Interestingly, the interaction effects (year by treatment) indicated that maize yield was not statistically different among T3 to T5 in the year 2015, but in the year 2016 and 2017, the maize yield was outperformed in T4 than T3 and T5. In 2017, even the maize yield was higher by 22% under 75% N with SDI (T3) over to 100% N with furrow irrigation (T5) and this was similar to 50% of N with SDI (T2, Table 1).

**Table 1:** Effect of N- fertigation through subsurface drip irrigation on maize, wheat and maize-wheat system grain yield, irrigation water use and irrigation water productivity in CA-based maize wheat system on permanent beds.

Parameters		Grain yield (Mg ha^–1^)			Irrigation water used (mm ha^-1^)			Irrigation water productivity (kg grain m^–3^)			
		Maize	Wheat	System	Maize	Wheat	System	Maize	Wheat		System
Year											
2015–16		7.73a	4.26c	12.0	108b	201c	309c	8.71a	2.21b		4.30a
2016–17		6.95ab	5.12a	12.07	111b	205b	316b	7.08b	2.57a		4.05a
2017–18		6.53b	4.80b	11.34	195a	230a	425a	3.53c	2.30b		2.89b
Treatment											
T1		3.78d	2.19e	5.98d	104c	152d	256d	4.49c	1.42d		2.49d
T2		7.29c	4.50d	11.79c	111b	175c	286c	7.55b	2.57c		4.25c
T3		7.93b	5.30c	13.23b	111b	181b	292b	8.15ab	2.93b		4.62b
T4		8.78a	5.97a	14.75a	111b	178bc	289bc	8.94a	3.35a		5.21a
T5		7.58bc	5.68b	13.26b	252a	374a	626a	3.08d	1.55d		2.17e
Year*treatment											
2015–16	T1	4.50j	1.39i	5.89	70f	116h	186i	6.46de		1.20g	3.17fg
	T2	7.97bcde	4.11g	12.08	75f	164g	240h	10.58ab		2.50e	5.04bc
	T3	8.41abcd	4.97de	13.38	76f	184d	260f	11.10b		2.70de	5.15b
	T4	9.10a	5.60c	14.7	77f	174ef	251g	11.88a		3.21abc	5.86a
	T5	8.68abc	5.24d	13.92	244b	365b	609b	3.56fg		1.44g	2.29hi
2016–17	T1	3.72jk	2.62h	6.34	72f	170fg	242h	5.17ef		1.54g	2.62gh
	T2	7.00ghi	4.82ef	11.81	87e	180de	267f	8.03cd		2.68de	4.42cd
	T3	7.68efgh	5.76bc	13.44	87e	180de	267f	8.82bc		3.20bx	5.03b
	T4	8.58ab	6.40a	14.98	87e	180de	267f	9.85ab		3.56a	5.61ab
	T5	7.76cef	6.01b	13.77	220c	317c	536c	3.53fg		1.90f	2.57gh
2017–18	T1	3.13k	2.56h	5.69	170d	170fg	340e	1.84g		1.51g	1.67ij
	T2	6.89fhi	4.58f	11.46	170d	180de	350d	4.05f		2.54de	3.27f
	T3	7.70deg	5.17d	12.87	170d	180de	350d	4.53ef		2.87cd	3.68ef
	T4	8.65ab	5.91bc	14.56	170d	180de	350d	5.09ef		3.28ab	4.16de
	T5	6.31i	5.78bc	12.1	293a	440a	733a	2.16g		1.32g	1.65j
ANOVA P value											
Year		0.025	0.002	0.058	< 0.001	< 0.001	< 0.001	< .001	0.005*		< 0.001
Treatment		< 0.001	< 0.001	< 0.001	< 0.001	< 0.001	< 0.001	< .001	< 0.001		< 0.001
Year*treatment		0.041	0.016	0.138	< 0.001	< 0.001	< 0.001	< .001	0.01		0.001

*Refer to Table 5 for treatment details. Means followed by a common letter or no letter within a column are not significantly different by the HSD-test (Tukey’s honestly significant difference) at the 5% level of significance.

### Wheat

Wheat grain yield, irrespective of treatments was significantly higher in 2016–17 (5.12 t ha^–1^) compared to 2017–18 (4.80 t ha^–1^) but decreased again in 2015–16 (4.26 t ha^–1^) (Table 1). In all the 3 years of study, wheat responded significantly to applied N up to 120 kg ha^–1^ (100% recommended N dose) under SDI in the maize-wheat system (Table 1). The increase in wheat yield (averaged over 3 years) was 173% under T4 compared to no N control (T1). There was no saving of N using SDI in wheat but the 3-yr mean results indicate that the SDI system helped to achieve a significantly higher yield by 5.1% in T4 compared to T5 (Table 1). In 2015–16 and 2016–17, wheat grain yield with T4 was significantly higher by 6.9 and 6.5% compared to T5 at similar fertilizer N rates, respectively, (Table 1) while wheat yield was similar under T4 and T5 in 2017–18. Interaction results (year by treatment) showed that the highest wheat grain yield was recorded under SDI with 100% N (T4) followed by furrow irrigation with 100% N (T5) in the year 2016–17. Unlike maize, SDI with 75% N (T3) performed inferior for wheat yield than T5, except in 2016–17, where both were found at par.

### System

Overall, the system yield of maize-wheat (MW) did not differ among the years (Table 1) unlike individual crops, this was compensated by maize and wheat yield. The 3-yr average data show that the total system MW system yield was significantly differed among the treatments except for T3 and T5. The highest system grain yield was under T4 which was higher by 2.0% compared to T3 and T5 (Table 1). On a system basis, the yield was similar under T3 and T5 resulting in saving of 25% of fertilizer N. There were no interaction effects observed on system yield and system yield ranged from 5.69 to 14.98 Mg ha^–1^.

### Irrigation water used and irrigation water productivity (WPi)

Total irrigation water applied in maize, irrespective of treatments was significantly higher in 2017 (195 mm) compared to 2016 (111 mm) and 2015 (108 mm) (Table 1). The N rates (T2-T4) showed a non-significant difference in the total amount of irrigation given in maize under SDI (Table 1). This was because all these treatments received the same amount of water in every irrigation applied. The average saving in irrigation water under the SDI system was 141 mm compared with the CT system and this was 56% lower compared to later (Table 1).

Similarly, total irrigation water applied in wheat, irrespective of treatments was significantly higher in 2017–18 (230 mm) compared to 2016–17 (205 mm) and 2015–16 (201 mm) (Table 1). The N rates (T3-T4) showed a non-significant effect on the total amount of irrigation given in wheat under SDI and were significantly higher than T1 and T2 treatments (Table 1). The average saving in irrigation water in wheat under the SDI system was 196 mm compared to the CT system. The quantity of irrigation water given to wheat in T4 was on an average 53.0% lower compared to T5 (Table 1).

Like maize and wheat crops, the higher amount of irrigation water was applied (425 mm) during 2017–18 and the lowest was in 2015–16 (309 mm). The saving in irrigation water input in the maize-wheat system was 54% in T4 compared to T5 during the study period (Table 1). The average saving in irrigation water in the maize-wheat system under the SDI system was 337 mm compared with the CT system (Table 1).

Interaction effects of the year by treatments results showed that the highest irrigation water was applied in T5 during 2017–18 for both maize and wheat crops and system level and it was lowest under T1 during 2015–16.

WPi of maize, irrespective of treatments was significantly higher in 2015 (8.72 kg m^–3^) compared to 2016 (7.08 kg m^–3^) and further decreased again in 2017 (3.53 kg m^–3^) (Table 1). The 3-yr mean data show that the WPi of maize was higher by 2.9 times in T4 compared to T5 (Table 1). In SDI treatment WP_i_ of maize in T4 ranged from 5.09 to 11.9 kg m^–3^ in comparison to 2.16 to 3.56 kg m^–3^ in T5 during 3 years of experimentation (Table 1). The WP_i_ of maize in T4 was significantly higher by 3.3, 2.8, and 2.4 times compared to T5 in 2015, 2016, and 2017, respectively (Table 1).

Like maize, WPi of wheat, irrespective of treatments was significantly higher in 2016–17 (2.57 kg m^–3^) compared to 2015–16 (2.21 kg m^–3^) and 2017–18 (2.30 kg m^–3^) (Table 1). The 3-yr mean data show that the WP_i_ of wheat was significantly higher by 2.2 times in T4 compared to T5 (Table 1). The WP_i_ of wheat in the SDI system (T4) ranged from 3.21 to 3.56 kg m^-3^ in comparison with 1.32–1.90 kg m^-3^ in T5 in the three-year experimentation (Table 1). The WP_i_ of wheat in T4 was significantly higher by 2.24, 1.87 and 2.48 times compared to T5 in 2015–16, 2016–17 and 2017–18, respectively (Table 1). WPi of the maize-wheat system, irrespective of treatments was significantly higher in 2015 (4.30 kg m^–3^) compared to 2016 (4.05 kg m^–3^) but decreased again in 2017 (2.89 kg m^–3^) (Table 1). The 3-yr mean data show that WP_i_ of the maize-wheat system was significantly higher by 2.4 times in T4 compared to T5 (Table 1). The system based WP_i_ in T4 was significantly higher by 2.56, 2.18, and 2.52 times compared to T5 in 2015, 2016 and 2017, respectively (Table 1).

### Grain N content, grain N uptake, and agronomic and N uptake efficiency

The grain N uptake of maize was similar in both years (Table 2). However, it was significantly higher (11.4%) in 2016–17 compared to 2017–18 in wheat (Table 2). The grain N uptake of maize and wheat increased significantly with the increasing rate of N under the SDI system (Table 2). In 100% N treatment with SDI (T4), grain N uptake was significantly higher by 30.2 and 15.6% (2-year mean) in maize and wheat compared to the conventional FI system (T5), respectively (Table 2). The SDI system did not show a significant effect on grain N uptake efficiency (NupE_G_) in maize and wheat at different N rates on the mean of two years’ data (Table 2). At the same N rate, NupE_G_ in maize was significantly (*p* < 0.05) higher by 44.0% in SDI (T4) than in T5 (Table 2). The corresponding increase in wheat grain NupE_G_ in T4 compared to T5 was 22.0%. The total N uptake of maize and wheat (2017–18) increased significantly with the increasing rate of N under the SDI system (Table 1s). In 100% N treatment with SDI (T4), total N uptake was significantly higher by 44.0 and 17.2% in maize and wheat compared to the conventional FI system (T5), respectively (Table 1s). The SDI system did not show a significant effect on NUE in maize and wheat (2017–18) at different N rates (Table 1s). In 100% N treatment with SDI (T4), NUE was significantly higher by 67.7 and 24.6% in maize and wheat compared to the conventional FI system (T5), respectively (Table 1s).

**Table 2:** Effect of subsurface fertigation and flood irrigation on grain N uptake, N uptake efficiency in grain (NupE_G_) and agronomic efficiency of nitrogen (AEN; kg grain kg^–1^ N applied) in maize and wheat in CA-based maize- wheat system.

Parameters		Grain N uptake (kg ha^–1^)		NupE_G_ (%)		AEN (kg grain kg^-1^ N-applied)	
		Maize	Wheat	Maize	Wheat	Maize	Wheat
Year							
2015–16		–	–	–	–	34.91	38.02a
2016–17		97.06	95.77a	64.95	84.25	34.53	32.83b
2017–18		96.03	85.96b	69.42	71.61	37.19	29.30c
Treatment							
T1		32.89d	30.58d	–	–	–	–
T2		91.26c	78.81c	77.83a	80.39a	46.69a	38.47a
T3		112.22b	103.69b	70.52a	81.24a	36.86b	34.52b
T4		139.33a	129.35a	70.96a	82.31a	33.30b	31.49c
T5		107.02b	111.89b	49.42b	67.76b	25.33c	29.06c
Year*treatment							
2015–16	T1	–	–	–	–	–	–
	T2	–	–	–	–	46.32	45.24
	T3	–	–	–	–	34.77	39.70
	T4	–	–	–	–	30.67	35.07
	T5	–	–	–	–	27.88	32.07
2016–17	T1	35.25	30.44	–	–	–	–
	T2	91.23	81.21	74.64	84.62	43.66	36.65
	T3	109.25	111.36	65.78	89.91	35.19	34.88
	T4	135.71	136.58	66.97	88.45	32.39	31.53
	T5	113.86	119.25	52.41	74.01	26.90	28.26
2017–18	T1	30.52	30.71	–	–	–	–
	T2	91.28	76.41	81.01	76.17	50.10	33.54
	T3	115.20	96.03	75.27	72.57	40.60	28.97
	T4	142.96	122.12	74.95	76.18	36.84	27.88
	T5	100.19	104.52	46.44	61.51	21.22	26.83
ANOVA P value							
Year		0.774	0.005	0.398	0.097	0.824	0.014
Treatment		< 0.001	< 0.001	0.001	0.038*	< 0.001	< 0.001
Year*treatment		0.099	0.112	0.282	0.844	0.349	0.108

*Refer to Table 5 for treatment details. Means followed by a common letter or no letter within a column are not significantly different by the HSD-test (Tukey’s honestly significant difference) at the 5% level of significance.

The agronomic efficiency of nitrogen (AEN) of maize and wheat generally decreased significantly (p < 0.05) with the increasing rate of N up to 75% of recommended N rate (T3). The 3-yr mean data showed that AEN in maize was significantly higher (31.5%) in T4 compared to T5 (Table 2). The AEN in wheat, irrespective of the treatments was significantly higher in 2015–16 compared to 2016–17 and 2017–18 (Table 2). The 3-yr mean data showed that AEN in maize was significantly higher (8.4%) in T4 compared to T5 (Table 2).

### Regression equations, optimum dose and economic optimum dose of N

The relationships between fertilizer N rates and grain yield of maize and wheat were best described by quadratic functions (Fig. 1s). The regression equations, regression coefficient, optimum dose (OD) of N and economic optimum dose (EOD) of N are presented in (Table 3). The OD of N to get maximum yield and EOD were 140 kg N ha^–1^ and 138 kg N ha^–1^ for maize, respectively (Table 3). The corresponding values for wheat were 225 kg N ha^–1^ and 221 kg N ha^–1^, respectively (Table 3).

**Table 3:** Regression equations, regression coefficients, optimum dose of nitrogen (OD) and economic optimum dose (EOD) in maize and wheat.

Crop	Regression equation	Regression coefficient (R^2^)	OD (kg ha^–1^)	EOD (kg ha^–1^)
Maize	− 0.0002x^2^ + 0.0561x + 3.806	0.99	140	138
Wheat	− 0.0001x^2^ + 0.0449x + 2.1929	0.99	225	221

### Mineral N

**Figure 3:**
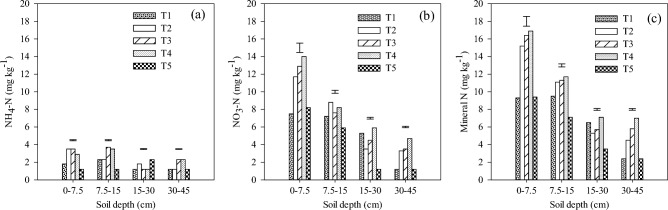
Effect of nitrogen rate and drip fertigation on (**a**) ammoniacal nitrogen (NH_4_–N); (**b**) nitrate nitrogen (NO_3_–N) and (**c**) mineral N at different soil depths after wheat 2017–18 in CA-based maize-wheat system. Vertical bars are standard error (SE) within each treatment. Refer to Table 5 for treatment details.

There was a significant difference in ammoniacal N content amongst different treatments at all the soil depths (Fig. 3a). The NH_4_-N content varied from 1.2 to 3.5 (0–7.5 cm), 1.2 to 3.7 (7.5–15 cm) and 1.2 to 2.3 mg kg^–1^ (15–30 and 30–45 cm) soil depths, respectively (Fig. 3a). The NH_4_-N content was significantly higher in T4 than that of T5 in 0–7.5 and 7.5–15 cm soil depths whereas it was significantly higher in T5 compared to T4 at 15–30 cm soil depth. Similarly, there was a significant difference in nitrate-nitrogen content amongst different treatments at all the soil depths (Fig. 3b). The NO_3_-N content varied from 7.5 to 14.0 (0.7.5 cm), 5.9 to 8.8 (7.5–15 cm), 1.2 to 5.9 (15–30 cm), and 1.2 to 4.7 mg kg^–1^ in 30–45 cm soil depth (Fig. 3b). NO_3_–N was significantly higher in T4 than that of T5. Similarly, higher mineral N content (NH_4_–N + NO_3_–N) was found under T4 compared to T5 at all the soil depths (Fig. 3c).

### Economic profitability

**Figure 4:**
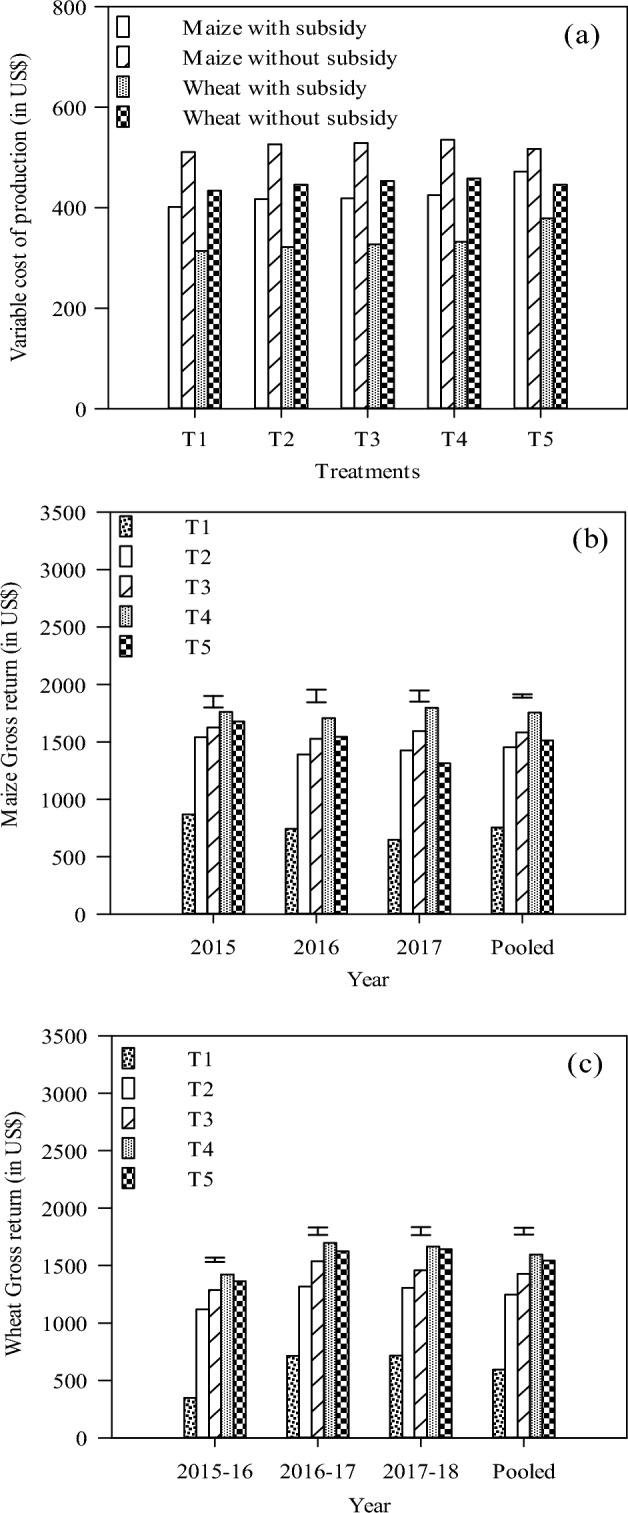
Variable cost of production (**a**) and gross return (in US$) in maize (**b**) and wheat (**c**) under different management practices on subsurface drip irrigation system. Vertical bars are standard error (SE) within each treatment. Refer to Table 5 for treatment details.

Economic profitability plays a pivotal role while considering crop management practices. Considering Scenario 1 with subsidy on electricity as well as 80% subsidy on a drip system, the variable costs of production in each of maize and wheat were lesser by US$ 47 in CA-based SDI plots (T4) than that under the conventional maize-wheat system (T5) (Fig. 4a). Under T5, the higher variable cost compared to SDI was due to higher labor and fuel costs for seedbed preparation in both maize and wheat. In Scenario 2, with no subsidy on electricity and drip system, the variable costs of production in maize and wheat were higher by US$ 18 and 12 in SDI treatment (T4) compared with T5, respectively (Fig. 4a). The high variable costs under T4 were primarily due to high drip costs. The gross returns in maize, irrespective of the treatments were significantly higher by 8.2 and 10.3% in 2015 compared with 2016 and 2017, respectively, (Fig. 4b). The 3-yr mean data show that gross returns in maize were significantly higher (16.1%) in T4 compared to conventional FI (T5) (Fig. 4b). The gross returns in wheat, irrespective of the treatments were significantly higher by 24.8% in 2016–17 compared to 2015–16 (Fig. 4c). The 3-yr mean data show that gross returns in wheat were significantly higher (3.4%) in T4 compared to T5 (Fig. 4c).

Considering Scenario 1 with a subsidy on electricity as well as 80% subsidy on a drip system, the net returns from the maize and maize-wheat system were non-significantly different in 3 years (Table 4). The net returns in wheat, irrespective of the treatments were significantly higher by 35.1% in 2016–17 than in 2015–16 (Table 4). Similar was the trend for net returns in maize, wheat and maize-wheat systems for Scenario 2 (Table 4). In Scenario 1, the 3-yr mean data show that net returns were significantly higher by 27.9, 8.5 and 17.6% in maize, wheat and maize-wheat system, respectively under T4 compared to conventional FI (T5) (Table  4). In Scenario 2, the corresponding increase was 22.6, 3.6 and 12.7% in maize, wheat and maize-wheat system, respectively, (Table 4).

**Table 4:** Economics analysis (net returns and B:C ratio) of maize-wheat system under different management practices with 80% subsidy and without subsidy on subsurface drip irrigation system.

Parameters		Net return (USD ha^–1^) with subsidy			Net return (USD ha^–1^) without subsidy			B:C ratio with subsidy		B:C ratio without subsidy		
		Maize	Wheat	MW system	Maize	Wheat	MW system	Maize	Wheat	Maize	Wheat	
Year												
2015–16		1068	773b	1841	971	660b	1631	2.49	2.28b	1.87		1.46b
2016–17		955	1044a	1999	859	931a	1790	2.22	3.09a	1.65		2.08a
2017–18		929	1023a	1952	832	910a	1742	2.17	3.03a	1.54		2.01a
Treatment												
T1		351d	279d	630d	242d	159d	401d	0.87d	0.89d	0.48c		0.36d
T2		1036c	925c	1962c	927c	801c	1729c	2.49bc	2.88c	1.76b		1.79c
T3		1164b	1100b	2264b	1054b	974b	2028b	2.78b	3.37b	1.99b		2.15b
T4		1330a	1263a	2593a	1220a	1137a	2357a	3.13a	3.80a	2.28a		2.48a
T5		1040c	1164b	2204b	995bc	1097a	2092b	2.20c	3.07c	1.93b		2.46a
Year*treatment												
2015–16	T1	467e	35	503	358e	− 85	274	1.16fg	0.11	0.71e		− 0.20
	T2	1124bcd	796	1920	1015bcd	672	1687	2.70bcd	2.48	1.96bcd		1.51
	T3	1207abc	958	2165	1097	832	1929	2.88bcd	2.93	2.11bcd		1.83
	T4	1336ab	1090	2426	1226ab	964	2190	3.14ab	3.28	2.32ab		2.11
	T5	1206abc	984	2189	1161abc	917	2077	2.56bcd	2.59	2.25abc		2.06
2016–17	T1	339e	401	740	230e	281	511	0.84g	1.28	0.46e		0.64
	T2	974cd	996	1970	865cd	872	1737	2.34cde	3.10	1.66cd		1.95
	T3	1108bcd	1211	2319	998bcd	1085	2083	2.65bcd	3.70	1.91bcd		2.40
	T4	1283ab	1366	2648	1173ab	1240	2412	3.02abc	4.11	2.21ab		2.71
	T5	1073bcd	1246	2318	1028bcd	1179	2206	2.27de	3.29	2.01bcd		2.70
2017–18	T1	246e	402	648	137e	282	419	0.61g	1.28	0.26e		0.64
	T2	1011bcd	984	1995	902cd	860	1762	2.43cde	3.06	1.67bd		1.92
	T3	1176abc	1132	2308	1066bcd	1006	2072	2.81bcd	3.46	1.97bcd		2.22
	T4	1371a	1332	2703	1261a	1206	2467	3.22a	4.01	2.30ac		2.63
	T5	841d	1264	2105	796d	1197	1993	1.78ef	3.33	1.52d		2.61
ANOVA P value												
Year		0.148	< 0.001	0.051	0.148	< 0.001	0.051	0.161	< 0.001	0.089		< 0.001
Treatment		< 0.001	< 0.001	< 0.001	< 0.001	< 0.001	< 0.001	< 0.001	< 0.001	< 0.001		< 0.001
Year*treatment		0.023	0.116	0.266	0.023	0.116	0.266	0.046	0.080	0.049		0.092

*Refer to Table 5 for treatment details. Means followed by a common letter or no letter within a column are not significantly different by the HSD-test (Tukey’s honestly significant difference) at the 5% level of significance.

Considering Scenario 1 with subsidy on electricity as well as 80% subsidy on a drip system, the benefit to cost (B:C) ratio for the SDI system was 3.47 compared to 2.64 for T5 (Table 4). In Scenario 1, the 3-yr mean data show that B:C ratio was significantly higher by 42.3 and 23.8% in maize and wheat, respectively under T4 compared to conventional FI (T5) (Table 4). Considering Scenario 2 without subsidy on electricity and drip system, the B:C ratio for the SDI system was 2.38 compared to 2.20 for T5 (Table 4). In Scenario 2, the 3-yr pooled data show that B:C ratio was significantly higher by 18.1% in maize under T4 compared to conventional FI (T5) (Table 4). The net returns and B:C ratio from SDI will still be more even when the subsidy is reduced to 25% from 80%.

## Discussion

### Effect of CA and fertigation on crop yields

The maize grain yield was markedly affected by the seasonal rainfall distribution pattern. For example, the high reduction in maize yield observed in 2017 under T5 was possibly owing to heavier rainfall (191 mm) received in August compared to 166 and 89 mm in 2015 and 2016, respectively, that adversely affected maize growth due to temporary flooding. Many researchers^8,26,27^ reported higher maize yield under permanent raised bed planting with residue mulching compared with CT fresh bed layout without mulch on fine-textured and sandy loam soils because maize under fresh beds system suffered from waterlogging due to soil crusting and poor soil aeration. The greater negative effect of flooding on maize yield in T5 may also be ascribed to the lower infiltration rate commonly observed in fresh beds CT system compared to CA-based maize-wheat system^19,28^. In contrast, Jat et al.^29^ and Ram et al.^30^ observed non-significant differences in grain yield of maize on permanent raised beds and fresh beds due to the high infiltration rate of loamy sand soil. In a recent study, Jat et al.^19^ recorded similar maize yield under flood irrigation and SDI systems but the latter received 25% less fertilizer N in CA-based maize-wheat-mung bean (*Vigna radiata* Lamm) system under the climatic conditions nearly similar to that of the present study. The maize performance also depends on the seasonal weather and climatic variations, in well distributed and normal rainfall year both systems perform similarly but under erratic and heavy rainfall-fall events and longer drought spells seasons the permanent beds perform better than fresh beds^8,26,27^. Lamm and Trooien^31^ and Tarkalson et al.^32^ from semi-arid regions of Nebraska, USA, reported similar or higher maize yield with SDI compared to flood irrigation because of the precise supply of water and N to the crop requirement. The higher grain weight in 2015 compared to 2017 contributed to a higher maize grain yield in the first year (Table 2s). However, there is no information available on grain yield with different N doses with SDI. The higher yield of maize under 100% N under SDI could be attributed due to N application in root zone through fertigation and deep placement also reduces the losses of N which also met the plant demand in more synchronized. The increase in wheat yield in 2016–17 could be due to favorable weather conditions compared with the first year. For example, maximum and minimum temperatures in March 2016–17 were lower by 1.5 to 4.4 °C compared with 2015–16 and 2017–18. Aryal et al.^33^ and Gupta et al.^34^ reported that weather is very significant in determining wheat grain yield under similar ecologies. Gupta et al.^34^ demonstrated an increase in wheat yield when the daily temperature in the particular year was 2.2–3.3 °C lower during the grain filling stage compared with other years in their study. The higher av. grain weight and grains per spike in 2016–17 compared to other years contributed to higher wheat grain yield in the second year (Table 2s). Our findings are consistent with the earlier researchers^16–19^ who reported similar or higher wheat yields under SDI compared to flood irrigation on medium to fine-textured soils. The higher total system WEY in the first and second years compared with the third year was due to either higher maize or wheat grain yield in the first and second years. The higher total system productivity (WEY) under SDI might be due to the beneficial effects of CA practices on crop growth and the better synchrony of split-N fertigation and crop N demand at various growth stages in both maize and wheat. Better supply of water and N through SDI resulted in higher yields of maize and wheat compare to conventional FI system due to reduction in N losses and improving availability of irrigation water. Similar observations were also concluded by Lu et al.^15^; Li et al.^35^ and Yolcu and Cetin^36^ under SDI in maize and wheat crops.

### Effect of CA and fertigation on irrigation water saving and water productivity

The higher saving in irrigation water under the SDI system compared with the CT system might be due to the uniform distribution of irrigation water in the root zone in SDI which minimizes evaporation and percolation losses and further additional benefits contributed from the crop residue mulching in permanent beds systems. Researchers^23,37,38^ reported large savings in irrigation water in SDI treatment in both crops of maize and wheat because of low drainage and evaporation losses. Our findings supported the results from earlier studies^19,23,31,39^ showing a 23 to 55% reduction in irrigation water use and increased WP_i_ compared to conventional FI. The increase in WP_i_ is ascribed to both increases in both crop yields and irrigation water saving. Earlier studies^19,23^ also showed a 32–53% reduction in irrigation-water use in wheat under SDI over conventional flood systems. Our results are consistent with the findings of Chen et al.^16^, Sidhu et al.^18^, and Jat et al.^19^ using SDI in wheat under rice–wheat and maize-wheat cropping systems. Crop residue retention on the surface in CA-based wheat and maize under SDI (T4) might also have helped in conserving soil moisture through a reduction in evaporation loss thereby reducing irrigation water requirement^29,37,38,40^.

### Effect of fertigation on N use efficiency

Our data showed markedly higher values of NupE_G_ in maize (67–75%) and wheat (76–88%) on permanent raised beds using SDI compared to CT flood irrigation system as 45–50% reported by Ladha et al.^41^. Similarly, higher NUE observed in maize (108%) and wheat (87%) on permanent raised beds using SDI compared to CT flood irrigation system (64–69%) in 2017–18. Bar-Yosef^42^ reported that NUE under SDI could be as high as 90% compared to 40–60% in conventional fertilizer application methods. Our data showed that there was a saving of 25% of N fertilizer in maize and system WEY due to the fact that N was applied in 5 equal splits in the SDI system in the root zone compared with broadcasting in 2 equal splits in the conventional FI system. The SDI system possibly reduced N losses via leaching, ammonia volatilization, and denitrification because small N doses were applied in several splits near the root zone along with small amounts of irrigation water^19,36^. According to Majeed et al.^10^ and Sandhu et al.^23^ the lower N losses in maize and wheat under permanent raised beds may lead to high recovery efficiency of applied N compared to conventional flat planting. The higher grain yields of maize and wheat under SDI resulted in significantly higher AEN under T4 compared to T5. The higher uptake of N under the SDI system was associated with greater biomass production and lower loss of applied fertilizer compared to conventional FI systems. Jat et al.^19^ and Sandhu et al.^23^ reported a significant increase in AEN and NUE under drip irrigation compared to the conventional FI systems in maize-wheat systems. Yadvinder-Singh et al.^21^ suggested that both timing and method of N application are important to increase NUE under straw mulched conditions. Li et al.^43^ reported that SDI increased the maize grain yield via increased plant nutrient uptake and reduced ammonia volatilization. Reduction in the use of N fertilizer via an increase in the NUE would lead to a substantial reduction in N_2_O emitted from the production and application of N fertilizers^44^. The OD of fertilizer N for maize in SDI was 140 kg ha^–1^ as compared to recommended N in conventional FI system (150 kg ha^–1^). Thus SDI system saved ~ 7% of fertilizer N with higher yield potential in maize. The EOD value for SDI system did not change significantly compared to OD due to low subsidized price of urea N. The OD and EOD values for wheat are higher compared to maize. The OD and EOD values for wheat are out of range of the experimental limits and thus will be erroneous to discuss here. The effect of fertigation in drip irrigation on NupE_G_ may be underestimated when irrigation water contains a significant amount of NO_3_-N because of the significant reduction in the use of irrigation water under SDI compared to conventional FI. Earlier studies^45–47^ reported a wide range of concentrations of NO_3_-N (0 to 40 mg N L^–1^) in the groundwater of NW India. However, irrigation water used in our study contributed a small amount of N (data not reported). The higher NO_3_-N in T4 than that of T5 may be attributed to the reason that water does not percolate in the lower depths under the SDI method because of light and frequent irrigations. Consistent with the results from our study, Yuvarajan and Mahendran^48^ reported higher available nutrients (NPK) in the 100% recommended fertilizer treatment in the post-harvest soil samples under SDI compared to the flood irrigation system in bananas.

### Effect of CA and fertigation on maize-wheat system profitability

Higher crop yields coupled with the low cost of tillage and labor resulted in the highest net returns in the SDI system (T4) compared with the conventional (T5) maize-wheat system. Considering Scenario 1 with a subsidy on electricity as well as 80% subsidy on a drip system, the higher gross and net returns in 2015 maize compared to other years were due to higher maize grain yield in 2015. Similarly, higher gross and net returns in 2016–17 wheat compared to 2015–16 wheat were due to higher wheat grain yield in 2016–17. The lower net returns in no-N control (T1) in 2015–16 wheat was due to low wheat grain yield (Table 1) coupled with less MSP of 2015–16 wheat (Sect. “Economic analysis”). In the rice–wheat system, Sidhu et al.^18^ reported a 29.8% higher net profit for the SDI system with 80% subsidy over the income of the conventional system (flood irrigation). Similarly, Jat et al.^19^ reported that SDI in a CA-based maize-wheat system provided 5.4% higher profitability over the conventional system (flood irrigation).

## Conclusions

This study showed that SDI can achieve crop yields similar to or higher than that of conventional flood irrigated and recommended N management practices in the sub-tropical regions. At the 100% recommended N rate, maize and wheat yields (mean for three yrs) were 15.7 and 5.2% higher under the SDI system than that in conventional FI treatment, respectively. Grain yield response of both maize and wheat to fertilizer N application was best described by quadratic response functions in SDI system. Irrigation WP under SDI was 183 and 120% higher for maize and wheat than that of conventional FI treatment, respectively. Similarly, NUE in the maize-wheat system significantly improved under SDI compared to the conventional FI systems with 25% saving of fertilizer N in maize. The net returns from the CA-based maize-wheat system layered with SDI provided 17.6% (with 80% subsidy on SDI) higher net returns than that for the conventional FI system. Overall, layering CA with SDI in a maize-wheat system resulted in a higher yield, WPi, NUE and net returns than the conventional FI, and can be adopted in other parts of South Asia. Future research should be conducted to understand N transformations in soil and quantify N loss mechanisms under SDI for designing effective fertigation strategies to further increase the NUE. The potential of using SDI for other nutrients (e.g. P, K and micronutrients) to achieve high productivity and nutrient use efficiency in the maize-wheat systems under different agro-climatic zones needs to be explored.

## Methods

### Experimental site

The experimental site at Borlaug Institute for South Asia (BISA), Ladhowal (30.99°N latitude, 75.44°E longitude, 229 m ASL), Punjab, India, which has a semi-arid sub-tropical climate. The hot, dry summer in March-June is followed by the monsoon season from late June-mid September and winter in October-February; 80% of the annual rainfall (average 734 mm) falls during the monsoon season. The soil texture (0–15 cm layer) is a Typic Haplustept with a loam (34% sand and 46% silt). The slope of the area is 0.2–0.4%. The values of pH (1:2 soil: water), electrical conductivity, Walkley–Black organic C, NaHCO_3_-extractable P, and NH_4_OAc-extractable K were 8.4, 0.53 dS m^–1^, 7.5 g kg^-1^, 21.8 mg kg^–1^, and 191 mg kg^–1^, respectively. The values of Zn, Fe, Mn and Cu were 1.35, 13.0, 2.25 and 1.52 mg kg^–1^, respectively. The contents of Ca, Mg and S were 80.0, 24.0 and 74.6 mg kg^–1^, respectively. The infiltration rate and water holding capacity were 6.75 mm h^–1^ and 23.3% (on a dry weight basis), respectively. At the start of the experiment, the bulk density of the 0–10 cm soil layer was 1.47 Mg m^-3^. The experiment was conducted for 3 years (2015–16, 2016–17, and 2017–18), and before this experiment, the site was under the maize-wheat system for the last three years.

### Measurement of rainfall and air temperature (T)

The daily rainfall, maximum temperature (Max T) and minimum temperature (Min T) were measured daily using a Davis weather station (Davis Vantage Pro 2 Weather Station) installed at the experimental site (Product Manual Davis Instrument Corp.).

### Experimental design and treatments

Raised bed planting system with 0.675 m wide beds (mid furrow to mid furrow) was established and the crops were planted on a 0.375 m wide top. After the wheat harvest in April 2014, the SDI system was set up in May 2014. To nullify the previous residual effects of maize-wheat treatments in experimental plots, general crops of maize (2014) and wheat (2014–15) were raised to stabilize the permanent raised beds before the start of the experiment in June 2015.

Five treatments were included in this study and details are given in Table 5. A randomized complete block design with 3 replicates was used in each season. The size of each plot was 81 m^2^ (2.70 m × 30 m).

**Table 5:** Treatment details.

Treatment number	Treatment	Maize N dose (kg N ha^–1^)	Wheat N dose (kg N ha^–1^)
T1	PB and SDI with + R and no–N control	0	0
T2	PB and SDI with + R and 50% of recommended fertilizer N	75	60
T3	PB and SDI with + R and 75% of recommended fertilizer N	112.5	90
T4	PB and SDI with + R and 100% of recommended fertilizer N	150	120
T5	FB for maize with furrow irrigation (FI) and CT flat for wheat with flood irrigation without residue and 100% of recommended fertilizer N	150	120

Where *PB* permanent beds, *SDI* subsurface drip irrigation, *N* nitrogen, *R* residue, *FB* fresh bed, *FI* furrow/flood irrigation, *CT* conventional till.

### Formation of raised beds

In June 2013, a seedbed for maize was prepared using a disc plough followed by two passes of the cultivator, and a laser-assisted land leveler to level the field. In the first week of June 2013, fresh raised beds were prepared using a 4-wheel tractor driven bed planter (National Agro Industries, Ludhiana, Punjab, India) and maize was sown on the top of beds using the recommended dose of fertilizers (120 kg N as urea + 26 kg P as di-ammonium phosphate + 50 kg K as muriate of potash ha^–1^) in the last week of June and harvested in the second week of October 2013. The fresh beds were maintained as such in designated plots for treatments T1 to T4 and termed as permanent raised beds for the next wheat crop. In T5, fresh beds were prepared in each season which was dismantled after harvest before sowing the next wheat crop. One row of maize was sown in the centre of the bed top at 20 cm plant to plant spacing, whereas 2 rows of wheat spaced at 20 cm were planted on each bed. In T5, beds, after the maize harvest was dismantled and conventional till wheat at 22.5 cm row spacing was planted on flatbeds. The permanent raised beds were never disturbed but the furrows were slightly reshaped once a year (before sowing of the maize crop) without disturbing the bed shape. A 4-wheel tractor with narrow tyres was used for all the operations to restrict movement only in furrows.

### Crop residue/stover management

In T1 to T4 treatments, maize at maturity was manually harvested above the cob height and removed from the plots, while the remaining ~ 50% portion of lower stubbles was retained which averaged 2.5, 4.6, 5.0, and 5.5 t ha^–1^ in T1, T2, T3, and T4, respectively Moreover, retaining 50% of maize stover on the field will help recycle more plant nutrients compared to that when the whole stover is removed. Total N addition through maize stubbles was 14, 32, 38, and 43 kg ha^–1^ in T1, T2, T3, and T4, respectively. Wheat was manually harvested at 20–22 cm above the ground level and straw stubbles were 0.8, 1.5, 1.8, and 2.0 t ha^-1^ in T1, T2, T3, and T4, respectively, as per N dose in different treatments. In conventional till treatment (T5), both maize and wheat crops were manually harvested at the ground level and all the biomass was removed from the plots.

### Installation of drip irrigation system

**Figure 5:**
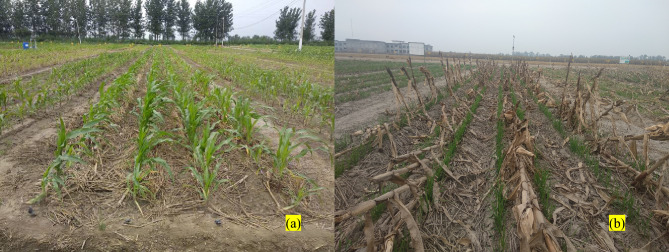
(**a**) Single row of maize and (**b**) two rows of wheat crop on each permanent bed with subsurface drip irrigation system.

The SDI system comprising polyethylene laterals (internal diameter 16 mm) was placed in the centre of each permanent raised bed. Based on the standardization and findings of the optimum depth of the SDI system (data not reported), for the ease of long-term tillage operations, 20 cm lateral depth was considered optimum for the maize-wheat system on permanent raised beds. The laterals used in this study had in-line emitters spaced at 30 cm with 2.0 L h^–1^ capacity at a pressure of 135 kPa for the entire wetting of a plot area. The lateral spacing was thus 67.5 cm apart (compatible with bed width) and placed at a depth of 20 cm (each lateral in the centre of each bed) for both maize and wheat (Fig. 5). Thus, each dripline served 1 row of maize and 2 rows of wheat on permanent raised beds. The subsurface driplines were laid using a drip laying machine^18^. Hydro cyclone filter and Venturi injector were used as per Sidhu et al.^18^.

Maize and wheat crops were irrigated using tube tensiometers monitored with a SoilSpec® vacuum gauge installed at 20 cm soil depth within each plot. Tensiometers installed as per Gupta et al.^34^ were regularly read each morning between 9:00 and 10:00 a.m. All plots received irrigation (70 mm in flood and 10 mm in SDI treatments) at the V8-10 stage (21–25 DAS) to all treatments of maize, and 70 mm in T5 before the second split of urea application at 40–45 DAS (if there was no rainfall event). Further irrigations were given whenever the average soil matric potential of the treatment plot decreased to – 50 ± 1 kPa. Common irrigation (70 mm in conventional flood and 10 mm in SDI treatments) was applied at the crown root initiation (CRI) stage (21-25 DAS) in all treatments of wheat, and 70 mm in conventional flood irrigation (T5) only before the second application of fertilizer dose. Like maize, additional irrigations were given whenever the average soil matric potential of the treatment plot decreased to − 35 ± 1 kPa. In the T5 treatment, PVC pipe was used to give irrigation. The irrigation amount was the same in all three replications of each treatment. Other crop management details are described below.

### Crop management

#### Maize

Fresh bed plots (T5) were irrigated with about 75 mm of water prior to tillage operations. Two passes of discing and one cultivator followed by two plankings were used for seedbed preparation for both maize and wheat at about 75% of field capacity moisture regime. The maize hybrid P3396 was sown on 17 July 2015, 18 June 2016, and 17 June 2017 using a seed rate of 20 kg ha^–1^ at a depth of 3–5 cm, using a double-disc planter fitted with an inclined plate seed metering mechanism (Dasmesh Mechanical Works, Punjab, India). A basal fertilizer dose of 26 kg P and 50 kg K ha^–1^ was drilled at planting using di-ammonium phosphate (single super phosphate in no N control) and muriate of potash, respectively. Total N applied was as per the N levels (T2-T5) of which 24 kg N ha^-1^ was applied as di-ammonium phosphate and the remaining N was applied as urea. In T5, the remaining (126 kg N ha^–1^) urea N was applied in 2 equal splits at 21–25 days after sowing (DAS) and 40–45 DAS. Under SDI, the remaining N as urea was applied in 5 equal split doses at 10-day intervals starting at 20 DAS, and the amount of urea was calculated as per the N levels in each treatment (T2 to T4). Maize crop was manually harvested on 25 October 2015, 06 October 2016, and 08 October 2017. Herbicide Atrataf 50WP (Atrazine @ 1.25 kg ha^−1^) was applied in all the treatments for controlling the broad leaf weeds. The pest management was done by using Decis (Deltamethrin) @ 200 ml ha^−1^ in all the treatments.

#### Wheat

In the conventional till plot (T5), after maize harvest, pre-irrigation (~ 75 mm) was given before the preparation of the seedbed for wheat. After dismantling fresh beds in maize, a conventional flat seedbed was prepared for wheat involving 2 passes each of discing, tilling, and planking. Wheat (HD 2967) was seeded at 100 kg ha^–1^ at 3–5 cm depth with a Turbo Happy Seeder^49^. The wheat was sown on 10 November 2015, 29 October 2016, and 31 October 2017, respectively. Like maize, wheat also received a similar basal dose of P and K. However, the total N applied was as per the N levels (T2-T5) of which 24 kg N ha^–1^ was applied as DAP and the remaining N was applied as urea except for no nitrogen plot where P was applied as a single super phosphate. In T5, the remaining (96 kg N ha^–1^) fertilizer N was applied before irrigation in 2 equal splits at CRI (21–25 DAS) and the maximum tillering stage at 40–45 DAS. Under SDI, N was given in five equal splits starting from 21 days of sowing and applied each at a 10-day interval as per the nitrogen treatments. The manual harvesting of wheat crops (about 10–12 cm above ground level) was done on 16 April 2016, 13 April 2017, and 16 April 2018. The post emergence herbicides Topik (clodinafop 15% WP)@400 g ha^−1^ and Algrip (metsulfuron)@25 g ha^−1^) were applied in all the treatments at 25–30 DAS. The pest management was done by using precautionary sprays of propiconazole@500 ml ha^−1^ and Dimethoate 30% EC @ 500 ml ha^−1^ in all the treatments.

### Observations

#### Irrigation water input and irrigation water productivity

The amount of water applied to each treatment was measured using a water meter (Dasmesh Mechanical Works, Punjab, India) fitted to the delivery pipe close to the trial area. The total irrigation input (mm) to each crop was determined. Irrigation water productivity (WP_i_) for maize and wheat was calculated as the ratio of grain yield to total irrigation water applied.

#### Grain yield

Grain yield (at maturity) was determined by manually harvesting an area of 8.1 m^2^ (4-beds and 3 m row length in bed systems and 12-rows and 3 m row length for flat wheat) in the centre of each plot at ground level. Grain moisture content was determined after drying the subsamples in an oven at 60 °C to a constant weight. Grain yield was adjusted at 14.5% (maize) and 12% (wheat) grain moisture and expressed as Mg ha^-1^. Average grain weight was based on the weight of 100 and 1000 grains selected from the threshed grain subsamples of maize and wheat, respectively. Average grains per cob/spike were determined from randomly selected 10 cobs/spikes. Spike density (m^-2^) in wheat was done at 2 locations (1 m row length) for each treatment at maturity. The total annual maize-wheat system yield was determined by adding the yield of maize to the yield of wheat.

#### Grain N uptake and N- use efficiency

At maturity, total N concentration in the grain subsamples in 2016–17 and 2017–18 and stover/straw subsamples in 2017–18 was determined by H_2_SO_4_-HClO_4_ digestion and analysis of digestate by micro Kjeldahl method. The NUE was calculated as under.

(i) The agronomic efficiency of applied N (AEN) was calculated as below:
  $${\text{AEN (kg grain/kg N applied) }} = \, \begin{array}{*{20}c} {\underline{{\text{(grain yield in N fertilized plot - grain yield in no N plot)}}} } \\ {\text{(quantity of N fertilizer applied in N fertilized plot)}} \\ \end{array} ,$$
(ii) Grain N uptake efficiency (NupE_G_) was calculated as:
  $${\text{NupEG (\% ) }} = \, \begin{array}{*{20}c} {\underline{{\text{(total grain N uptake in N fertilized plot - total N uptake in no N plot)}}} } \\ {\text{(quantity of N fertilizer applied in N fertilized plot)}} \\ \end{array} \times 100,$$

for calculating grain N uptake efficiency in T5 treatment, N uptake for no N plot (T1) was used presuming small differences in N uptake between SDI and conventional till plots.

(iii) N use efficiency (NUE) based on total N uptake was calculated as:

Total N uptake in both grain and stover/straw was determined to calculate NUE similar to that explained for NupE_G_ as above.

Irrigation water containing low concentrations of NH_4_-N (0.30 mg L^–1^) and NO_3_-N (0.40 mg L^–1^) hence, the irrigation N input data were not considered for calculating NUE.

#### Grain yield response to fertilizer N and calculation of optimum N doses

While quadratic response equations (Y = a + bx + cx^2^) were developed for maize and wheat using SigmaPlot 15.0.where Y is grain yield (Mg ha^-1^) of maize or wheat; x is rate of N (kg ha^–1^); and a, b, and c are constants of quadratic response equation.

Optimum N dose and economic optimum N dose were calculated from the quadratic response equations as described by Fausti et al.^50^. The market value of the cost of N as urea was taken as US$ 0.15 kg^-1^ and the prevailing market prices of maize and wheat in 2017–18 were US$ 195 Mg^–1^ and US$ 238 Mg^–1^, respectively.

#### Ammoniacal and nitrate–N in soil

Soil samples were collected at wheat harvest in 2017–18 from all the experimental plots from 0–7.5, 7.5–15, 15–30, and 30–45 cm depths. Mineral N was extracted from the fresh soil samples using 2* M* KCl solution. Micro-Kjeldahl steam distillation method was used for the determination of NH_4_-N and NO_3_-N in 2* M* KCl extracts^51^. The data were corrected for soil moisture content.

### Economic analysis

To calculate the economics of the SDI system, partial budgeting was done as explained by Sidhu et al.^18^. The net profits were the extra income that resulted from using the SDI system in our partial budget. The cost of operations and inputs (variable costs) to grow the crop are given in Table 6. In the economic analysis, the fixed costs such as land value and interest were not included. The prices used were the government guaranteed minimum support prices (MSP) of maize (182, 187 and 195 US$ Mg^-1^ in 2015, 2016 and 2017, respectively) and wheat (209, 223 and 238 US$ Mg^-1^ in 2015–16, 2016–17 and 2017–18, respectively). This budget sheet was prepared as referred by the Department of Economics, Punjab Agricultural University (PAU), Ludhiana, India (Table 6). The number of laborers was calculated considering 8 working hours to be equivalent to 1 person-day required for all agronomic practices as detailed in Sidhu et al.^18^. Similarly, the time (h) required by a tractor-drawn machine/implement to complete a field operation such as tillage, seeding, and harvesting was recorded and expressed as h ha^−1^. To calculate the irrigation cost, initial and final reading units on the electric meter were noted after running the tube well for 10 min in both SDI and flood irrigated plots. The price of 1 kW of electricity is taken as US$ 0.11. The time taken to complete 1 cm and 5 cm of irrigation in SDI and flood irrigated plots were 53 min and 60 min, respectively. For irrigation costs, the electricity charges were calculated as US$ 2.15 and US$ 8.22 ha-cm^–1^ for drip and flood, respectively. Irrigations number ranged from 7 to 17 for maize and 17 to 18 for wheat under SDI during the 3-year study. Labor cost per person-day was US$ 4.79. The cost of drip irrigation, its life span, and depreciation cost were the same as explained in Sidhu et al.^18^. The gross returns included income from the sale of grain and straw of both maize and wheat crops. The gross returns were calculated by using the market minimum support price (MSP) for both maize and wheat grains (Table 6). The calculations of net return (NR) were done as per Sidhu et al.^18^. In Scenario 1 (with subsidy), the parameters considered were electricity and 80% subsidy on a drip system whereas in Scenario 2 (without subsidy), there was no subsidy on electricity and drip system. One US$ was equivalent to Indian Rs. 73/- based on the exchange rate.

**Table 6:** Cost of key inputs and outputs used for economic analysis in maize-wheat cropping systems.

Particulars	Amount (US$)
Price of maize seed	3.84 kg^–1^
Price of wheat seed	0.48 kg^–1^
Urea	0.07 kg^–1^
Di-ammonium-phosphate (DAP)	0.33 kg^–1^
Preparation of conventional till wheat plots	75.3 ha^–1^
Sowing of maize	34.2 ha^–1^
Sowing of wheat	24 ha^–1^
Seed treatment in maize	0.82 ha^–1^
Seed treatment in wheat	1.92 ha^–1^
Weedicide in maize	6.33 ha^–1^
Weedicide in wheat	16.1 ha^–1^
Pesticide in maize	5.48 ha^–1^
Pesticide in wheat	8.66 ha^–1^
Cost of irrigation in SDI	0.003/m^2^/irrigation
Cost of irrigation in flood	0.004/m^2^/irrigation
Labour cost	4.38 day^–1^
Combine harvester and straw reaper in wheat	92.5 ha^-1^
Combine harvester in maize	85.6 ha^–1^
Market price of wheat straw	34.2 Mg^–1^
*Drip cost for 67.5 cm spacing (with 80% subsidy and 15-year life)	21.0 ha^–1^
Drip cost for 67.5 cm spacing (without subsidy and 15-year life)	104.8 ha^–1^

1US$ = 73.0 Indian rupees (Rs).

*Drip cost is same for both maize and wheat.

All methods were performed in accordance with the relevant guidelines and regulations.

### Statistical analysis

The normality of data was tested using the SAS 9.2 software package (SAS Institute, Cary, NC) and the normality assumption of ANOVA was fully met, there was no need for data transformation. In this study, we followed the procedure to build statistical models for data analysis for randomized complete block design (RCBD) in fixed plots, which is suggested for fixed plot experiments with the complexity of tillage and management: treatment and year, together with within-year replication^52^. The analysis advanced using a complete two-factor analysis of variance (ANOVA). The treatments were fixed effects and were randomly allocated to plots. ‘Year’ was a repeated factor; this was combined with the treatment model by introducing the term “Year + Treatment x Year”. ‘Replication’ was a randomized unit, so we kept it under random effect by including replication interactions with all treatments. The final model was tested using the JMP software.

$${\text{Fixed effect}}:{\text{ Year }} + {\text{ Treatment }} + {\text{ Treatment }} \times {\text{ Year,}}$$

$${\text{Random effect}}:{\text{ Replication }} + {\text{ Replication }} \times {\text{ Year}}{.}$$

All variable means were compared using Tukey’s honest significant difference at p = 0.05, where significant treatment means were separated using alphabet letters.

## Supplementary Information

Supplementary Information.

## Data Availability

The datasets used and/or analysed during the current study are available from the corresponding author on reasonable request.
