# Supplementary material for: Precise irrigation water and nitrogen management improve water and nitrogen use efficiencies under conservation agriculture in the maize-wheat systems
Source: Sci Rep. 2023 Jul 26;13:12060. doi: 10.1038/s41598-023-38953-6 (PMC10372093; doi:10.1038/s41598-023-38953-6)
Supplement: Supplementary file 1 — Supplementary Information. [file 41598_2023_38953_MOESM1_ESM.doc]

**Table 1s:** Effect of N- fertigation through subsurface drip irrigation on total N uptake and NUE in maize and wheat (2017-18) in CA-based maize wheat system.

| ***Treatment** | **Maize** | | **Wheat** | |
| --- | --- | --- | --- | --- |
|  | **Total N uptake (kg ha^-1^)** | **NUE (%)** | **Total N uptake (kg ha^-1^)** | **NUE (%)** |
| T1 | 51.85d | -- | 35.71d | -- |
| T2 | 139.3c | 116.6a | 86.93c | 85.4a |
| T3 | 171.8b | 106.6a | 110.4b | 83.0a |
| T4 | 213.1a | 107.5a | 139.5a | 86.5a |
| T5 | 148.0c | 64.1b | 119.0b | 69.4b |

* Refer to Table 1 for treatment details.

**Table 2s:** Effect of N- fertigation through subsurface drip irrigation on yield attributes of maize and wheat in CA-based maize wheat system on permanent beds.

| **Year** |  | **Maize 100-grains weight (g)** | **Maize grains cob^-1^** | **Wheat Av. grain weight (g)** | **Wheat grains per spike (no.)** | **Wheat spike density (m^-2^)** |
| --- | --- | --- | --- | --- | --- | --- |
| 2015-16 |  | 28.0a | 445b | 39.2ab | 46b | 287b |
| 2016-17 |  | 27.6a | 420c | 39.6a | 47a | 321a |
| 2017-18 |  | 22.2b | 465a | 39.1b | 46b | 323a |
| *Treatment | |  |  |  |  |  |
| T1 |  | 22.5d | 306c | 38.1c | 37b | 187e |
| T2 |  | 26.0c | 467b | 39.7ab | 48a | 287d |
| T3 |  | 27.2ab | 472b | 39.8a | 48a | 332c |
| T4 |  | 27.4a | 521a | 40.2a | 49a | 366b |
| T5 |  | 26.5bc | 452b | 38.7bc | 48a | 380a |
| Year*Treatment | | | | | | |
| 2015-16 | T1 | 25.2 | 329 | 37.3 | 34c | 141h |
|  | T2 | 27.6 | 463 | 39.8 | 48a | 279f |
|  | T3 | 28.9 | 467 | 39.8 | 48a | 309e |
|  | T4 | 29.6 | 515 | 40.4 | 49a | 350cd |
|  | T5 | 28.6 | 453 | 38.6 | 49a | 358bcd |
| 2016-17 | T1 | 24.4 | 289 | 38.7 | 38b | 201g |
|  | T2 | 28.0 | 440 | 39.9 | 49a | 290ef |
|  | T3 | 29.1 | 439 | 40.1 | 49a | 344d |
|  | T4 | 28.5 | 492 | 40.7 | 50a | 378ab |
|  | T5 | 28.2 | 439 | 38.7 | 49a | 394a |
| 2017-18 | T1 | 18.0 | 300 | 38.3 | 38b | 219g |
|  | T2 | 22.5 | 498 | 39.3 | 48a | 291ef |
|  | T3 | 23.6 | 509 | 39.4 | 47a | 344d |
|  | T4 | 24.2 | 555 | 39.5 | 48a | 371abc |
|  | T5 | 22.8 | 465 | 38.8 | 47a | 389a |
| ANOVA P Value | | | | | | |
| Year |  | <0.001 | 0.005 | 0.049 | 0.014 | 0.001 |
| Treatment | | <0.001 | <0.001 | <0.001 | <0.001 | <0.001 |
| Year*Treatment | | 0.102 | 0.275 | 0.408 | 0.001 | <0.001 |

* Refer to Table 1 for treatment details.

**Fig. 1s:** Regression between fertilizer N application rates and average grain yield of maize (a) and wheat (b)
